# Immunosuppression in Renal Transplantation and Dyslipidemia, Which Factors Should Be Considered?

**DOI:** 10.5812/numonthly.14189

**Published:** 2013-11-13

**Authors:** Alfredo De Giorgi, Fabio Fabbian

**Affiliations:** 1 Department of Medical Science, University of Ferrara, University Hospital S. Anna, Ferrara, Italy

**Keywords:** Dyslipidemias, Kidney Transplantation, Immunosuppression

***Dear Editor,***

Secondary dyslipidemia is the most common form of lipid metabolism derangement, due to the fact that primitive dyslipidemia represent only 0.5-2% of cases (1).

Transplantation is a common cause of dyslipidemia, it has been shown that it defines metabolic syndrome, a common finding in renal transplant recipients (RTR) (2), and statins are within the top 15 drugs used by these patients (3). Moreover the increasing prescription of statins in this population is related to the frequent screening for dyslipidemia in transplanted patients (3). Immunosuppression could cause hypercholesterolemia and Cyclosporine (CsA) is widely used as immunosuppressive drug even if in the recent years physicians prescribe more frequently Tacrolimus than CsA (3). Moreover renal function in RTR is reduced and in many causes they are long-term treated with steroids.

An additional risk factor for dyslipidemia should be taken into account in RTR such as chronic kidney disease, a condition in which triglycerides are increased and HDL-cholesterol is reduced. The main causes of these alterations are increased plasma levels of VLDL, IDL, LDL, Lp(a) and reduced activity of hepatic triglyceride lipase and peripheral lipoprotein lipase (4).

Hypercholesterolemia secondary to steroids is due to hyperinsulinemia and peripheral insulin resistance increases hepatic synthesis of VLDL, moreover steroids reduce the release of ACTH with up-regulation of LDL-cholesterol hepatic receptors (5).

On the contrary, the mechanism responsible for hypercholesterolemia during CsA treatment is still a matter of debate. CsA is a lipophilic drug and is carried by HDL and LDL cholesterol; it has been suggested that the drug impairs hepatic clearance of LDL (6). Moreover hepatic sterol regulatory element binding protein-2 is activated leading to increased VLDL synthesis, and secretion of bile and bile salts is impaired causing reduced cholesterol elimination (7).

Hosseini et al. (8) study evaluated the relationship between CsA and dyslipidemia in RTR; they showed that plasma levels of cholesterol and triglycerides started increasing 4-12 months after beginning of immunosuppressive therapy, while subsequently HDL-cholesterol decreased and LDL-cholesterol increased. Logistic regression analysis showed that female gender and serum creatinine were the major risk factors for hypercholesterolemia and hypertriglyceridemia; on the contrary, CsA was not related to them. Similar results were shown by Laufer et al. (9) in heart transplant recipients. In their study the main risk factors were the history of cardiac disease leading to transplantation and steroids therapy. Hricik et al. (10) evaluated RTR and showed that reduction of steroid’s dose caused decreasing total plasma cholesterol levels.

Cardiovascular disease is a major problem in RTR due to its relationship with morbidity and mortality and graft loss. Statins seem to have a protective effect in RTR, especially on risk of major cardiac events and they need to be considered during patients follow-up (4).
